# Cytotoxic edema and diffusion restriction as an early pathoradiologic marker in canavan disease: case report and review of the literature

**DOI:** 10.1186/s13023-016-0549-1

**Published:** 2016-12-07

**Authors:** Steven T. Merrill, Gary R. Nelson, Nicola Longo, Joshua L. Bonkowsky

**Affiliations:** 1 College of Osteopathic Medicine, Touro University Nevada, Henderson, NV USA; 2 Division of Pediatric Neurology, University of Utah School of Medicine, Salt Lake City, UT USA; 3 Division of Medical Genetics, Department of Pediatrics, University of Utah School of Medicine, Salt Lake City, UT USA; 4 Department of Pediatrics, University of Utah School of Medicine, 295 Chipeta Way/Williams Building, 84108 Salt Lake City, UT USA

**Keywords:** Leukodystrophy, Canavan disease, MRI, Diffusion restriction, Cytotoxic edema

## Abstract

**Background:**

Canavan disease is a devastating autosomal recessive leukodystrophy leading to spongiform degeneration of the white matter. There is no cure or treatment for Canavan disease, and disease progression is poorly understood.

**Results:**

We report a new presentation of a patient found to have Canavan disease; brain magnetic resonance imaging (MRI) revealed white matter cytotoxic edema, indicative of an acute active destructive process. We performed a comprehensive review of published cases of Canavan disease reporting brain MRI findings, and found that cytotoxic brain edema is frequently reported in early Canavan disease.

**Conclusions:**

Our results and the literature review support the notion of an acute phase in Canavan disease progression. These findings suggest that there is a window available for therapeutic intervention and support the need for early identification of patients with Canavan disease.

## Background

Canavan disease is a devastating autosomal recessive leukodystrophy leading to spongiform degeneration of the white matter [1]. Deficient aspartoacylase activity from mutations in *ASPA* lead to accumulation of N-acetylaspartic acid (NAA) in the brain, myelin loss [2, 3], and NAA acidemia with elevated levels of NAA present in cerebrospinal fluid, blood, and urine [4]. Patients with Canavan disease typically present with developmental delay, focal neurological signs, macrocephaly, and even neurological deterioration in the first year of life [5]. There is no cure or treatment for Canavan disease, and disease progression is poorly understood. Magnetic resonance imaging (MRI) of the brain can be diagnostic, based on the presence of diffuse symmetric T2 hyperintense signal in the cortical white matter and basal ganglia with an accompanying elevated NAA peak seen on magnetic resonance spectroscopy (MRS) [6, 7].

We report a patient presenting with brain MRI white matter cytotoxic edema, indicative of an acute active destructive process, who was found to have Canavan disease. Review of the literature suggests that cytotoxic edema is a common finding in early Canavan disease, and supports the potential for a therapeutic window of intervention.

## Methods

A ten-week old boy presented with episodes of right pendular horizontal nystagmus lasting two to three seconds. He had been born full term following an uncomplicated pregnancy to nonconsanguineous Caucasian parents. Vitals and general physical exam were unremarkable and head circumference was at the 63^rd^ percentile. Neurologic examination was notable for a lack of visual tracking, central hypotonia, limb hyperreflexia, and choreiform limb movements.

We conducted PubMed searches for the terms “Canavan” and “MRI” or “Canavan” and “diffusion” to identify potential relevant publications. Identified publications were reviewed for relevant cases involving cytotoxic edema on brain MRI and data were extracted (STM and JLB).

## Results

**Fig. 1:**
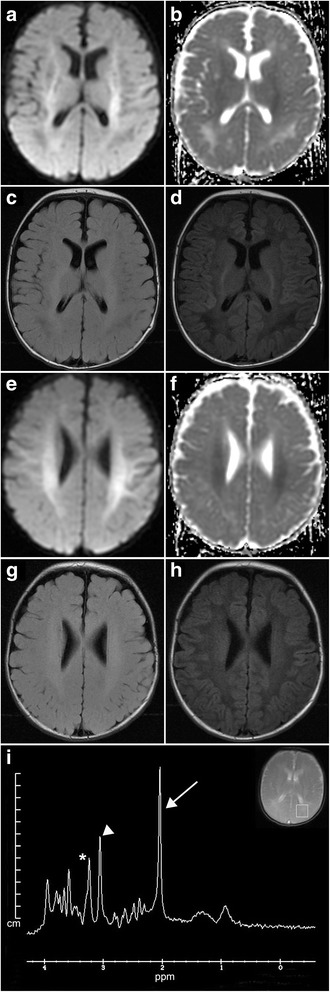
**a-h** Axial MRIs. **a** DWI shows hyperintensity in the internal capsule and subcortical white matter; B) ADC demonstrates corresponding hypointensities; **c** T2 FLAIR image; **d** T1 image. **e** DWI with hyperintensities in the subcortical white matter; **f** Corresponding ADC hypointensities; **g** T2 image; **d** T1 image. **i** Single voxel MRS demonstrates increased NAA (arrow, peak at 2.02 ppm; choline, asterisk, 3.2 ppm; creatine, arrowhead, 3 ppm); inset shows area of analysis in the posterior left hemisphere

Brain MRI performed at presentation revealed extensive diffusion restriction symmetrically in the subcortical white matter of both cerebral hemispheres, extending inferiorly in the corticospinal tracts and into the brainstem including the dorsal and ventral pons, with T2 hyperintensity and T1 hypointensity of the white matter (Fig. 1 a-h). Single voxel magnetic resonance spectroscopy (MRS) of the white matter of the posterior left hemisphere demonstrated an elevated peak at 2.02 ppm indicating increased NAA (Fig. 1i). The short echo-time (TE) spectrum N-acetylaspartic acid/creatine (NAA/CR) ratio was elevated at 2.45 (normal for age 1.71) [8]. Urine organic acids had elevated NAA excretion (>750 mmol NAA/mol creatinine), consistent with Canavan disease. Sequencing of the *ASPA* gene demonstrated a homozygous p.Ala305Glu (c.914 C > A) point mutation confirming the diagnosis.

To determine if cytotoxic edema with diffusion restriction was a common pathophysiological feature of Canavan disease we reviewed cases using PubMed. We identified 81 potentially relevant publications; including our own report there were 13 Canavan disease patients in whom cytotoxic edema and diffusion restriction was reported (Table 1). The median age of presentation was 12 months (average age 32 months; range 0.8 to 171 months). For the eleven patients in whom gender was reported, ten were male. Of nine cases reporting head size, one patient was microcephalic, five were normocephalic, and four were macrocephalic.

**Table 1:** Canavan disease patients reported with cytotoxic edema

Reference	Gender	Age (months)	Seizures	Delay	Hypotonia	Spasticity	Macrocephaly	Affected brain structures	Abnormal DWI brain structures
a	m	17	na	na	na	na	na	sc	sc
b	m	15	+	+	–	+	+	bs, cer, p, t, gp	bs, cer, p, t, gp
c	m	72	na	na	na	na	na	sc, cc	sc, cc
c	m	12	na	na	na	na	na	sc, ppv	sc
d	m	11	+	+	–	–	+	sc, bs	sc, bs
e	m	10	–	+	+	–	+	sc, gp, t	sc, gp, t
f	na	17	+	+	–	+	–	sc, gp, t, cer	sc, gp, t, cer
f	na	171	–	+	–	+	microcephaly	sc	sc
g	m	0.8	na	na	na	na	+	sc, bs, t, cer, bg	sc, bs, t, bg
h	m	7	na	na	na	na	na	sc	sc
i	f	84	–	+	–	–	–	bg, sc	bg, sc
j	m	4	+	–	–	–	–	bg, sc	bg, sc
k	m	2	–	–	–	–	–	bs, sc, intcap	bs, sc, intcap

References: a, Engelbrecht V, Scherer A, Rassek M et al. Diffusion-weighted MR imaging in the brain in children: findings in the normal brain and in the brain with white matter diseases. *Radiology*. 2002;222(2):410–8; b, [6]; c, Patay Z. Diffusion-weighted MR imaging in leukodystrophies. *Eur Radiol*. 2005;15(11):2284–303; d, Srikanth SG, Chandrashekar HS, Nagarajan K et al. Restricted diffusion in Canavan disease. *Childs Nerv Syst*. 2007;23(4):465–8.; e, Unalp A, Altiok E, Uran N et al. Novel mutation of aspartoacylase gene in a Turkish patient with Canavan disease. *J Trop Pediatr*. 2008;54(3):208–10.; f, Cakmakci H, Pekcevik Y, Yis U et al. Diagnostic value of proton MR spectroscopy and diffusion-weighted MR imaging in childhood inherited neurometabolic brain diseases and review of the literature. *Eur J Radiol*. 2010;74(3):e161-71; g, Rodrigues K, Grant PE. Diffusion-weighted imaging in neonates. *Neuroimaging Clin N Am*. 2011;21(1):127–51, viii; h, Perlman SJ, Mar S. Leukodystrophies. *Adv Exp Med Biol*. 2012;724:154–71; i, Nguyen HV, Ishak GE. Canavan disease – unusual imaging features in a child with mild clinical presentation. *Pediatr Radiol*. 2014;45:457–60.; j, [9]; k, this report

*Abbreviations*: *m* male, *f* female, *na* not available, *intcap* internal capsule, *bs* brain stem, *cc* corpus callosum, *bg* basal ganglia, *cer* cerebellum, *p* pons, *t* thalamus, *gp* globus pallidus, *ppv* parietal periventricular, *sc* subcortical white matter

## Discussion

We found that cytotoxic edema with corresponding diffusion restriction on brain MRI is more commonly observed in Canavan disease than previously appreciated. Besides our own case, we identified twelve other published reports of Canavan disease with diffusion restriction. It is difficult to assess the frequency of diffusion restriction in Canavan disease, since many published cases of Canavan disease do not include MRI reports, and diffusion restriction may only be present early in the disease course. Because diffusion restriction has not been considered as a presenting feature of Canavan disease, patients have sometimes first been evaluated for other non-leukodystrophy causes, e.g., stroke [9]. Further, it is increasing recognized that there is a broader phenotypic variability [5] with milder clinical courses in some patients [10] and even absence of features consider pathognomonic; for example the absence of macrocephaly [11].

Disease pathology in Canavan is related to NAA accumulation [2, 3]. In turn, the degree of ASPA impairment from different mutations correlates with clinical disease severity [12]. While NAA accumulation appears responsible for the spongiform myelin loss, NAA also has important roles in normal CNS function. Acetate derived from catabolism of NAA by ASPA is utilized by oligodendrocytes to synthesize the lipid component of the myelin sheath [1]. The ASPA enzyme is expressed widely in the body, but NAA is found exclusively in the brain [13, 14]. It has been shown that during neuronal development increased myelination correlates with increased ASPA activity [15]. NAA also appears to function as a molecular water pump in neurons [16]. These roles of NAA may contribute to the histopathology of Canavan disease, which includes astroglial swelling and intramyelinic edema [17, 18], and which may in turn be correlated with alterations in anisotropy and the diffusion coefficient seen on MRI. Diffuse brain edema with an increase in cerebral water content is also seen in other disorders (for example Congenital Muscular Dystrophy with Merosin Deficiency [19]), but without NAA accumulation, raising the possibility that NAA accumulation is not the primary cause of the edema.

Interest in improved understanding of the pathology and the early diagnosis of Canavan disease has arisen in part because of the potential for new treatments [20]. Gene therapy replacement of ASPA has been used successfully in a rodent Canavan model and appears safe in humans [21, 22]. Pharmacologic strategies to target osmotic pressure in Canavan also appear promising [23]. However, screening for Canavan disease is only recommended for high-risk groups [5], although advances in next-generation sequencing and in liquid chromatography-tandem mass spectrometry (e.g., [4]), suggest increased potential for broader newborn screening. The presence of cytotoxic edema, indicated by an increase in diffusion restriction [24], suggests that there is an acute neurotoxic process occurring. Because the diffusion restriction is abnormal for only approximately a week or less after damage, the presence of diffusion restriction shows that at least some of the disease pathology in Canavan disease is occurring post-natally. If a therapy was available it could be instituted as soon as the diagnosis was made, even if some disease progression had already occurred.

## Conclusions

Our case and review of the literature show that cytotoxic edema with diffusion restriction on brain MRI is often observed in infant-onset Canavan disease, and if an infant presents with diffusion restriction Canavan disease should be considered in the differential. Our results support the notion of an acute phase in disease progression. Together with evidence that NAA levels are related to myelin degeneration [2, 3], that NAA levels rise during the first year of life in Canavan disease [25], and the possibility for potential treatments [20, 22, 23], these findings suggest that there is a window available for therapeutic intervention and support the need for early identification of patients with Canavan disease.
